# Advanced Sustainable Approaches for the Valorization of High-Value Bioactive Compounds from Agro-Industrial Waste: Challenges and Opportunities

**DOI:** 10.3390/foods15091486

**Published:** 2026-04-24

**Authors:** Ivan M. Savic, Ivana M. Savic Gajic

**Affiliations:** Faculty of Technology, University of Nis, Bulevar Oslobodjenja 124, 16000 Leskovac, Serbia

The global transition towards sustainable and high-quality food systems is a central goal of advanced food science, driven by the need to improve resource circularity and reduce the ecological footprint of production [1]. A key component of this transition is the identification and application of naturally derived bioactive compounds—including polyphenols, lipophilic vitamins, essential minerals, and antioxidant complexes—as functional alternatives to synthetic additives [2]. This shift is further supported by increasing evidence of potential adverse effects associated with synthetic constituents, as well as growing consumer demand for clean-label and health-promoting formulations in both human and animal nutrition [3].

A major research focus is the upcycling of agro-industrial by-products, which are increasingly recognized as dynamic biochemical matrices rather than inert waste streams [4] and whose valorization is essential for circular bioeconomy development. However, recovery efficiency is strongly influenced by compositional variability and ongoing biotic transformations. The investigation of spent mushroom substrate (Contribution 1) represents a shift in the perception of agro-industrial residues, transitioning from viewing them as static waste streams to recognizing them as dynamic, evolving biochemical systems, thus highlighting that fungal cultivation is not merely a production process for fruiting bodies but a catalyst for a comprehensive reconfiguration of the substrate’s molecular landscape. Untargeted metabolomic analysis of *Pleurotus djamor* and *Pleurotus ostreatus* substrates revealed a significant depletion of low-molecular-weight compounds, including native polyphenols. This reduction is likely attributable to the intense enzymatic degradation or metabolic assimilation dictated by the fungal mycelium. However, the true significance of this study lies in the selective remodeling of the matrix, while certain essential bioactive constituents are reduced. The process facilitates a concomitant enrichment of specialized metabolites with high industrial potential, such as cerebroside B and ganoderenic acid D. These findings fundamentally challenge the traditional approach to waste valorization due to specific metabolites either remained stable or were newly synthesized. Collectively, this suggests that biotic transformations are highly specific to both the substrate and the species. For the industrial sector, this implies that agro-industrial waste can no longer be treated as a uniform or inert feedstock but must be managed as a living bioreactor. To harness this potential, a mechanistic understanding of these biotic transformations is essential, requiring the integration of high-resolution analytical platforms that can help map the precise “metabolic window”, which is the point where the target compound concentration reaches its peak. By optimizing the recovery of bioactive compounds based on the biological age and metabolic state of the matrix, it is possible to produce value-added products of higher quality. These products can meet strict standards for functionality and support scalability within the circular bioeconomy, as well. This approach effectively transforms an environmental liability into a strategic, high-value resource for the nutraceutical and functional food industries. The transition toward circular bioeconomy models depends on the efficient recovery of bioactive compounds from agri-food by-products, thus elevating waste valorization from a sustainability goal to a technological requirement [5]. These side-streams represent a significant source of functional ingredients capable of reducing environmental impact while meeting industrial performance criteria. Recent studies demonstrate their applicability across different systems, particularly as alternatives to synthetic additives; for example, onion solid waste has been used for the recovery of polyphenols and anthocyanins (Contribution 2). The extracts obtained using optimized pressurized liquid extraction provided oxidative stability in complex emulsions comparable to butylated hydroxytoluene. This supports the feasibility of replacing synthetic antioxidants with natural, upcycled alternatives without compromising product quality. In addition to technological functionality, agri-food by-products also contribute to nutritional enhancement. The incorporation of apple, sour cherry, and peach pomace into bakery products (Contribution 3) significantly improves their mineral and micronutrient content. This dual functionality, both technological and nutritional, represents an important advancement in functional food design. It links waste valorization with the development of nutritionally enriched products.

To further improve resource efficiency, current strategies increasingly rely on integrated processing systems, where agri-food by-products are utilized as substrates for cascading valorization. Green walnut husk represents a promising lignocellulosic residue in this context (Contribution 4). This study employed an optimized ultrasound-assisted extraction protocol combined with microwave pretreatment to enhance the recovery of antioxidant compounds. Process parameters, including extraction time, extraction temperature, and time of microwave pretreatment, were systematically optimized using response surface methodology based on a Box–Behnken design. Under optimal conditions, total phenolic content reached 3.69 g gallic acid equivalents per 100 g of dry matter. In addition to polyphenols, UHPLC–ESI–MS/MS analysis identified a wide range of compounds, including lipids, quinones, terpenoids, and organic acids, highlighting the chemical complexity of the extract. Importantly, this approach extends beyond single-step extraction. After bioactive recovery, the remaining biomass was further processed for cellulose isolation. Structural and hydration analyses demonstrated favorable functional properties, supporting its potential use in food and material applications. Such cascading strategies enable comprehensive biomass utilization and support the implementation of circular economy and zero-waste concepts.

The bioactive compound recovery from natural matrices remains a key technological challenge in food science, as conventional extraction methods are widely used but often limited by high solvent consumption, long processing times, low selectivity, and the risk of thermal degradation [6]. As a result, there is a growing need to adopt intensified and more sustainable extraction technologies. Techniques such as pulsed electric fields, supercritical fluid extraction, and membrane-based processes offer improved efficiency and environmental performance, although their industrial application is often constrained by scalability and cost [7]. Among these approaches, ultrasound-assisted extraction has emerged as a versatile and scalable option [8]. Its combination with other intensification techniques, such as microwave pretreatment, can significantly enhance mass transfer and extraction yield [9]. For example, the sequential application of microwaves and ultrasounds improved antioxidant recovery from green walnut husks (Contribution 4); similarly, ultrasound-assisted extraction has been successfully applied to *Spirulina platensis* (Contribution 5), enabling the simultaneous recovery of multiple bioactive fractions, including polyphenols, proteins, and pigments. A key aspect of these approaches is process optimization. Response surface methodology, particularly Box–Behnken design, enables systematic evaluation of process variables and their interactions (Contributions 2 and 4), thus allowing for the identification of optimal conditions by reducing experimental effort and resource use. Overall, these findings highlight the potential of hybrid and intensified extraction systems for sustainable recovery of bioactive compounds.

Advanced analytical methodologies are essential for the identification, quantification, and characterization of bioactive compounds, as well as for monitoring product quality and stability [10]. These tools enable a better understanding of the relationships between composition, processing conditions, and functional properties, supporting the development of consistent and standardized products. High-resolution techniques such, as HPLC-DAD and LC-MS/MS are particularly important for the characterization of complex matrices. They have been effectively applied to the profiling of phenolic compounds in onion waste extracts (Contribution 2) and green walnut husks (Contribution 4). Their high sensitivity and selectivity ensure reliable qualitative and quantitative analysis. In parallel, rapid and non-destructive techniques are gaining importance. Near-infrared spectroscopy combined with artificial neural networks has shown strong predictive performance for key properties of *Spirulina platensis* extracts, enabling efficient process monitoring (Contribution 5). Complementary techniques, including ATR-FTIR and colorimetric analysis, provide further insight into material properties, as demonstrated in studies on hazelnut cultivars (Contribution 6). Together, these approaches enhance analytical efficiency and support industrial applicability.

Despite their potential, many bioactive compounds exhibit limitations, such as low solubility, poor stability, and limited bioavailability [11]. These factors restrict their practical application in food systems. Therefore, strategies aimed at improving stability and delivery are essential. Encapsulation, nanoemulsions, and related delivery systems can enhance compound protection and enable controlled release [12]. In addition, the preservation of bioactive compounds begins at the extraction stage, and the selection of extraction conditions (temperature and solvent type) is necessary to minimize degradation and increase the stability of bioactive compounds [13]. This approach is illustrated by Bozinou et al. (Contribution 2), who combined pressurized liquid extraction with encapsulation to stabilize polyphenols from onion waste. Spray drying with maltodextrin and gum arabic was used as a protective matrix, improving stability and functionality in a model food system, such as salad dressing.

Future research should be focused on the discovery of new bioactive compounds and the identification of alternative natural sources, particularly in the context of global environmental change. Climate-related factors are expected to influence the availability and composition of plant-derived compounds. They affect both the supply of raw materials and the performance of final products [14], so the development of resilient and adaptive production systems will be necessary. As shown in this Special Issue, integrating advanced extraction technologies, high-resolution analytical methods, and sustainable resource management is a promising strategy for sustainable agri-food systems.

## Data Availability

Not applicable.
